# Bright sky-blue fluorescence with high color purity: assembly of luminescent diphenyl-anthracene lutetium-based coordination polymer†

**DOI:** 10.1039/d0ra10795f

**Published:** 2021-02-10

**Authors:** Yuichi Kitagawa, Ayu Naito, Koji Fushimi, Yasuchika Hasegawa

**Affiliations:** Faculty of Engineering, Hokkaido University Kita-13, Nishi-8 Sapporo Hokkaido 060-8628 Japan y-kitagawa@eng.hokudai.ac.jp hasegaway@eng.hokudai.ac.jp; Institute for Chemical Reaction Design and Discovery (WPI-ICReDD), Hokkaido University Sapporo Hokkaido 001-0021 Japan; Graduate School of Chemical Sciences and Engineering, Hokkaido University Kita-13, Nishi-8 Chome Sapporo Hokkaido 060-8628 Japan

## Abstract

Pure sky-blue fluorescence (FWHM: 50 nm) from lutetium-based coordination polymer with diphenyl-anthracene derivative is demonstrated for the first time. The observed high color purity is based on the tightly packed crystal structure of the coordination polymer with multiple CH–F interactions.

## Abstract 2

Pure sky-blue fluorescence (FWHM: 50 nm) from lutetium-based coordination polymer with diphenyl-anthracene derivative is demonstrated for the first time.

## Graphical abstract

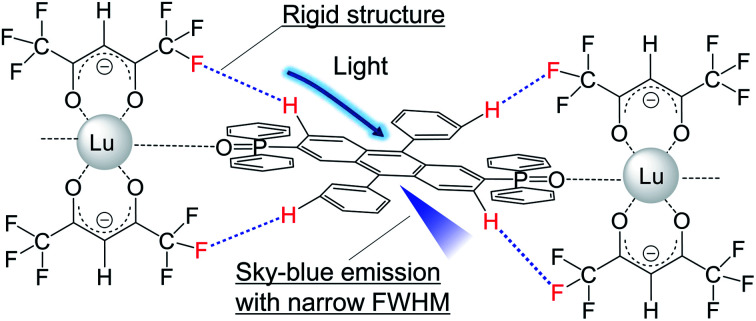

Organic luminophores are promising materials with strong light absorption and high emission quantum yield, which have been studied for development of display, illuminations, and fluorescence imaging.^1^ Among all the reported organic luminophores, the diphenyl-anthracene (DPA) derivatives have been used for the development of blue emissive materials used for fabricating organic light-emitting diodes (OLEDs) and sensors.^2^ Liu reported efficient DPA-based OLEDs possessing strong blue emission properties (brightness > 6600 cd m^−2^).^2*a*^ Colsmann reported the green-to-blue photon up-conversion process using a DPA-porphyrin hybrid system.^2*b*^ Vacha reported the effective photon up-conversion emission properties of DPA using Ag plasmon.^2*c*^ DPA-based luminophores need to be developed further to improve the purity of emission color. Controlled structural motion in the excited state can induce emission with high color purity.

Herein, we report the design of a DPA incorporated trivalent lanthanide coordination polymer. The multiple coordination sites assist the development of rigid and thermostable structures.^3^ The characteristic thermostable structure is expected to induce a fixation system in the excited structure, resulting in pure color emission. The DPA incorporated trivalent lanthanide coordination polymer can be potentially used for inducing emission with high color purity.

We prepared a novel coordination polymer containing Lu(hfa)_3_ (hfa: hexafluoroacetylacetonate) and phosphine oxide bridges with a DPA framework in its structure ([Lu(hfa)_3_DPA-P]_*n*_; DPA-P: (9,10-diphenyl-anthracene-2,6-diyl)bis(diphenylphosphine)oxide; Fig. S1†) as a proof of concept. The non-luminescent Lu(iii) ion was the lanthanide ion of choice used for linking the organic fluorophore. The hfa unit and the phosphine oxide ligand containing the DPA unit can potentially lead to the formation of multiple inter- or intramolecular CH–F interactions in the Lu(iii) coordination polymers (Fig. 1),^3*b*,*c*^ leading to suppressed excited motion. The heavy metal effect exhibited by the Lu(iii) ion can be suppressed in the presence of phosphine oxide, an electronic spacer.^4^ The Lu(iii) coordination polymer exhibited bright sky-blue emission with a narrow bandwidth (full width at half maximum (FHWM) = 50 nm). This width was smaller than that of DPA-P (FWHM = 77 nm). This novel approach of achieving pure color emission can potentially help in gaining insight into the design of organic luminophores.

**Fig. 1:**
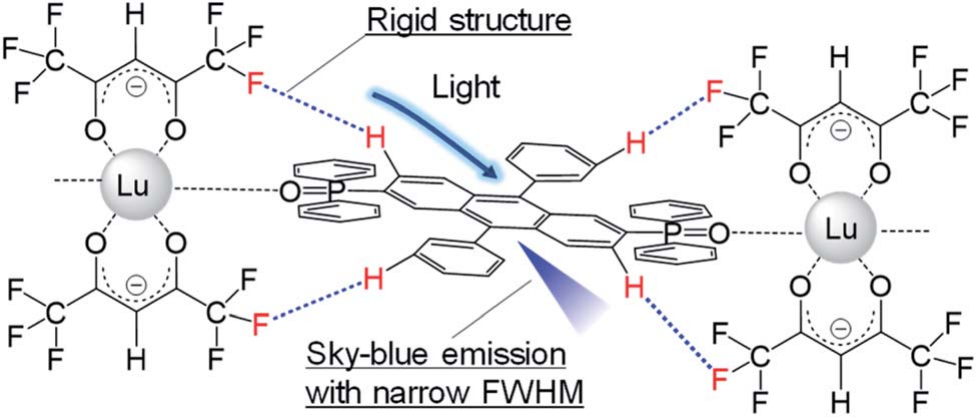
Schematic illustration of molecular design.

The single crystals of the DPA-P ligand and the [Lu(hfa)_3_DPA-P]_*n*_ complex were prepared by a recrystallization process using the CH_3_OH/CH_2_Cl_2_ solvent system. The crystal structures are shown in Fig. 2a and b. The DPA-P crystal belongs to the *P*2_1_/*n* space group (Table S1, ESI†). Intermolecular hydrogen bonding (O–H bonding) is present between the DPA-P units. The crystal of [Lu(hfa)_3_DPA-P]_*n*_ belongs to the *P*2_1_/*c* space group (Table S1, ESI†). The coordination site being present in the Lu(iii)-based coordination polymer unit comprises of three hfa ligands and one phosphine oxide ligand containing the DPA fluorophore. Inter- and intramolecular CH–F interactions (<3.0 Å) between the hfa unit and the aromatic DPA-P ligands were observed in the [Lu(hfa)_3_DPA-P]_*n*_ complex (Fig. 2b). 8-Coordinated Lu(iii) ions exhibiting a square antiprism structure (point group = *D*_4d_) was observed (ESI† for details).^5^ Thermogravimetric analysis (TGA) of the sample was performed. The TGA profiles of DPA-P and [Lu(hfa)_3_DPA-P]_*n*_ units are shown in Fig. 2c. The decomposition temperature of [Lu(hfa)_3_DPA-P]_*n*_ was 340 °C, which was higher than the decomposition temperature of the DPA-P ligand. The high thermostability of the system is attributed to the three-dimensional CH–F networks incorporated with DPA units.

**Fig. 2:**
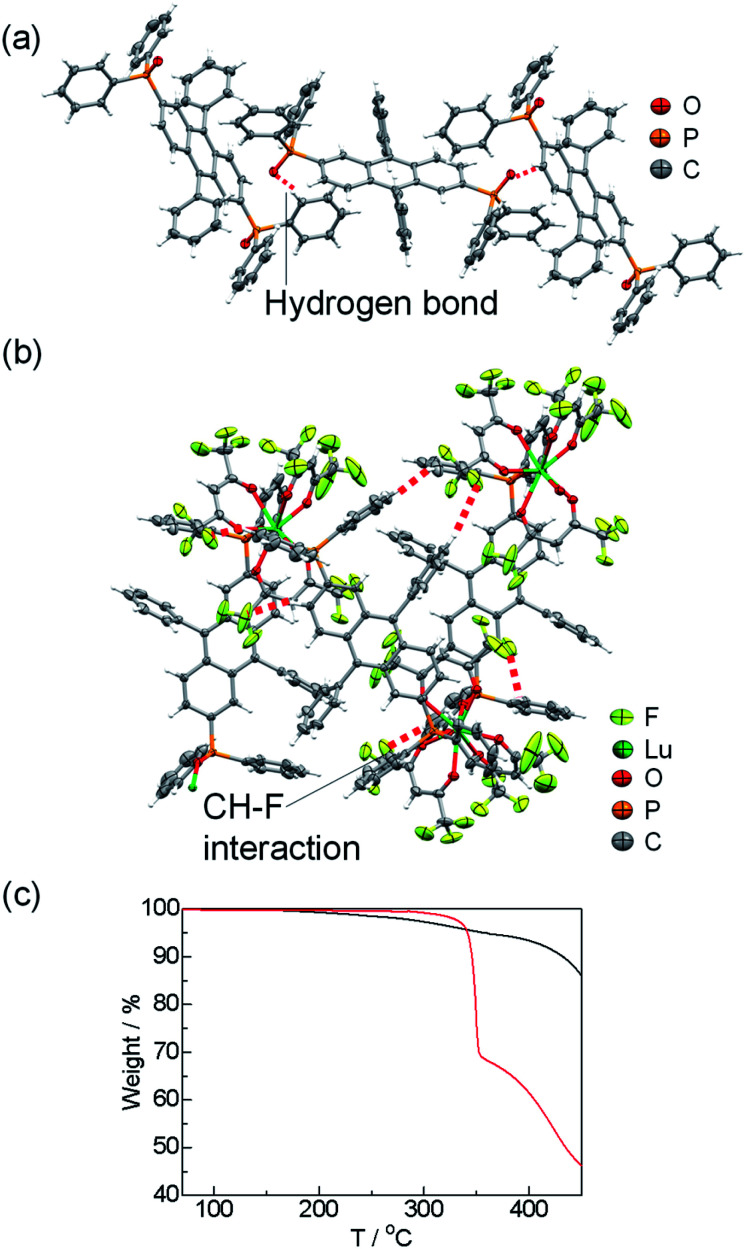
X-ray crystal structures of the DPA-P ligand (a) and the [Lu(hfa)_3_DPA-P]_*n*_ (b). TGA profiles (c) of the DPA-P ligand (black line) and the [Lu(hfa)_3_DPA-P]_*n*_ (red line).

The electronic absorption spectrum of the DPA-P ligand is shown in Fig. 3 (black broken line). The absorption bands at 368 nm, 386 nm, and 407 nm are attributed to the DPA-P unit. Incorporation of DPA into the Lu(iii)-based coordination polymer induced a slight red-shift (368 nm, 394 nm, and 416 nm), indicating a change in the electronic structure of the polymer. The emission peak of DPA-P at 466 nm (Fig. 3, solid black line) is blue-shifted due to ligand fixation (450 nm, solid red line). We observed a significant decrease in the FWHM (from 77 nm (DPA-P) to 50 nm ([Lu(hfa)_3_DPA-P]_*n*_)). This can be attributed to the effective molecular fixation into the three-dimensional CH–F network. This focus of this study is completely different from the previous study, which largely focuses on the enhancement of metal emission properties by organic ligands.^6^

**Fig. 3:**
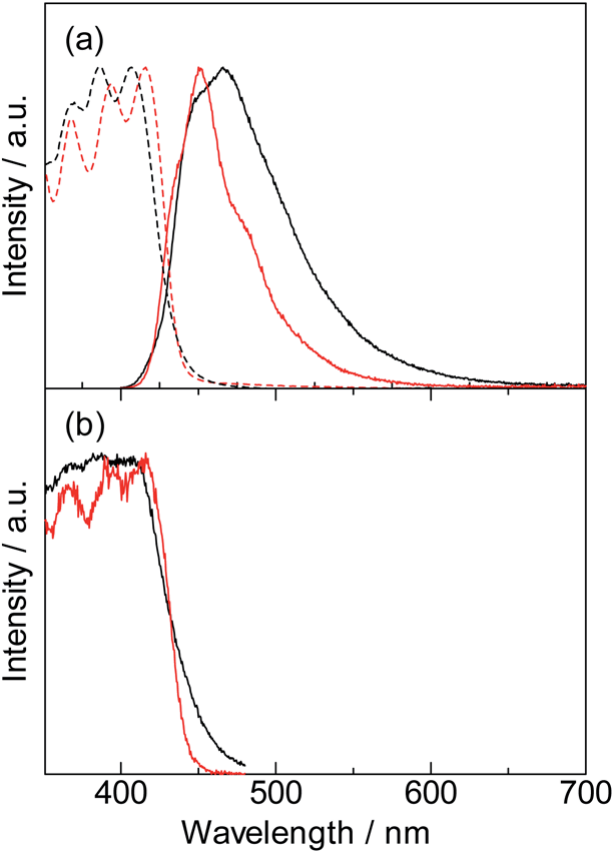
(a) Absorption (broken line), emission (solid line), and (b) excitation (solid line) spectra of DPA-P (black line) and [Lu(hfa)_3_DPA-P]_*n*_ (red line) in their solid states. The samples were 3000-fold dilution using KBr.

The photophysical parameters are summarized in Table 1. The emission quantum yield is also enhanced due to fixation (DPA-P: 18%, [Lu(hfa)_3_DPA-P]_*n*_: 25%). The radiative and non-radiative constants are estimated from the emission quantum yield and emission lifetime values. The radiative rate constant calculated for [Lu(hfa)_3_DPA-P]_*n*_ (fixation system) was larger than the radiative rate constant estimated for the DPA-P ligand. The increased radiative rate constant indicated the disruption of the symmetric electronic structure^7^ by coordination with multiple inter- or intra-molecular interactions. The non-radiative rate constant calculated for [Lu(hfa)_3_DPA-P]_*n*_ was comparable to the non-radiative rate constant calculated for DPA-P. These photophysical parameters provide the enhancement of emission quantum yield.

**Table 1:** Photophysical properties of DPA-P and [Lu(hfa)_3_DPA-P]_*n*_

	FWHM/nm	*τ* a/ns	*Φ* b/%	*k*_r_ c/s^−1^	*k*_nr_ d/s^−1^
DPA-P	77	4.5	18	4.0 × 10^7^	1.8 × 10^8^
[Lu(hfa)_3_DPA-P]_*n*_	50	3.6	25	6.9 × 10^7^	2.1 × 10^8^

a Emission lifetime (*λ*_ex_ = 380 nm) under Ar.

b Emission quantum yield (*λ*_ex_ = 380 nm) under Ar.

c Radiative rate constant.

d Non-radiative rate constant.

The emission mechanism is illustrated in Fig. 4. The emission stokes shift of the fixation system ([Lu(hfa)_3_DPA-P]_*n*_: 900 cm^−1^) was smaller than that of DPA-P (1910 cm^−1^). The shifts were estimated using the spectral fitting technique (Fig. 5). The small changes in the ground state and excited state electronic structures of the Lu(iii)-based coordination polymer can be attributed to the effective molecular fixation. The slight shift in the nuclear coordinate resulted in red-shifted absorption bands and blue-shifted fluorescence spectrum. This also resulted in the formation of narrow emission bands.

**Fig. 4:**
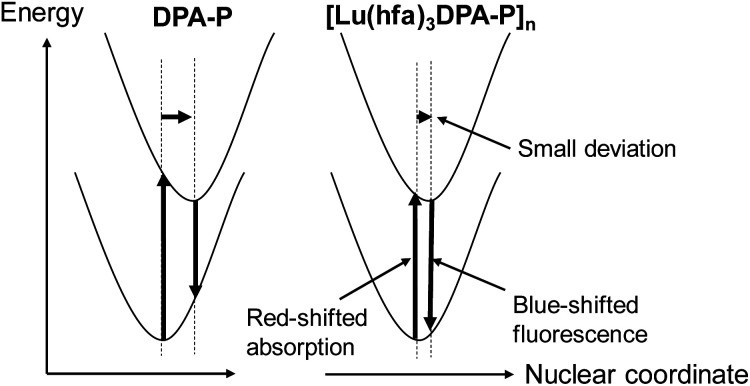
Energy diagram of DPA-P and DPA-P units present in the Lu(iii) coordination polymer.

**Fig. 5:**
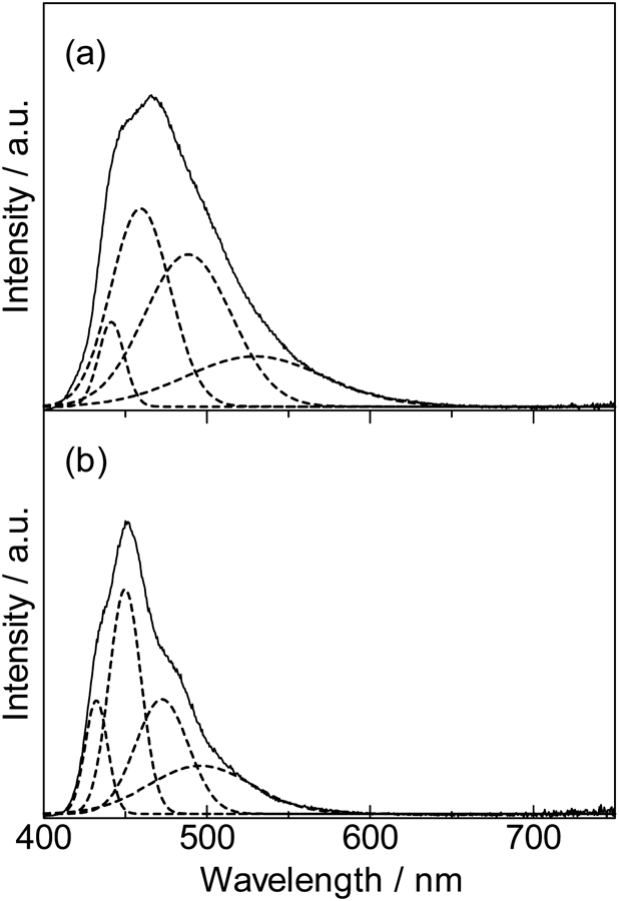
Band deconvolution analysis of DPA-P (a) and [Lu(hfa)_3_DPA-P]_*n*_ (b) emission spectra.

In conclusion, we successfully reported the first demonstration of bright, pure sky-blue emission of DPA derivative using the coordination polymer fixation. The fixation effectively suppresses the excited structural motion. The presented method opens up routes for the development of strong luminophores exhibiting high color purity.

## Conflicts of interest

There are no conflicts to declare.

## Supplementary Material

RA-011-D0RA10795F-s001

RA-011-D0RA10795F-s002
