# Supplementary material for: Bright sky-blue fluorescence with high color purity: assembly of luminescent diphenyl-anthracene lutetium-based coordination polymer
Source: RSC Adv. 2021 Feb 10;11(12):6604–6. doi: 10.1039/d0ra10795f (PMC8694909; doi:10.1039/d0ra10795f)
Supplement: RA-011-D0RA10795F-s001 [file RA-011-D0RA10795F-s001.pdf]

Electronic Supplementary Material (ESI) for RSC Advances.
This journal is © The Royal Society of Chemistry 2021

S1. Experimental section

General Methods: ^{1}H-NMR spectra were recorded in $CDCl_3$ on a JEOL ECS-400 (400 MHz) spectrometer; $CHCl_3$ (δ_H = 7.26 ppm) was used as the internal reference. Electrospray ionization (ESI) mass spectrometry was performed using JEOL JMS-T100 LP instrument. Elemental analyses were performed using MICRO CORDER JM10. Luminescence spectra for DPA-P and $[Lu(hfa)_3DPA\text{-}P]_n$ were measured using a Horiba FluoroLog®3 spectrofluorometer. Emission quantum yields for DPA-P and $[Lu(hfa)_3DPA\text{-}P]_n$ were measured using a FP-6300 spectrofluorometer with an integration sphere (ILF-533). The emission lifetimes were measured by a streak camera system (Streakscope C10627, Hamamatsu). Diffuse-reflection spectra were recorded with a JASCO V-670 spectrophotometer equipped with an integrating-sphere unit (JASCO ISN-723).

Materials: Magnesium sulfate, anhydrous (>98.0% (titration)) and chloroform-d_1 (99.8%) were purchased from Kanto Chemical Co., Inc. Potassium acetate (Guaranteed Reagent) and lutetium(III) acetate tetrahydrate (99.9 %) was purchased from Wako Pure Chemical Industries, Ltd. 2,6-Dibromo-9,10-diphenylanthracene (> 98.0%), diphenylphosphine (> 90.0 % (GC)), and palladium(II) acetate (> 98.0%) were purchased from Tokyo Chemical Industry Co., Ltd.

Preparation of [9,10-diphenyl-anthracene-2,6-diyl)bis(diphenylphosphine) oxide (DPA-P, Fig. S1, Scheme 1)]: A solution of potassium acetate (333 mg, 1.8 mmol) and palladium acetate (3.0 mg, 0.013 mmol) were added dropwise to a solution of 2,6-dibromo-9,10-diphenylanthracene (500 mg, 1.02 mmol) in dry DMA (15 mL). Diphenylphosphine (0.5 ml, 2.04 mmol) was then added to the solution, which was subsequently stirred for 24 h at 100°C. Then 120 mL of water was added to the solution and the obtained powder was filtrated. The product powder was dissolved in CH_2Cl_2 (100

mL) and a 30% H_2O_2 aqueous solution (2 mL) was added to the solution. The reaction mixture was stirred for 5 h. The product was extracted using $CHCl_3$ and washed with distilled water. The solvent was then evaporated to produce a powder. The recrystallization was performed using CH_2Cl_2/MeOH solvent (Yield: 210mg, 28%).

^{1}H-NMR (400 MHz, $CDCl_3$) δ/ppm = 7.91 (d, 2H, J = 16.0 Hz), 7.79 (dd, 2H, J = 11.6 Hz), 7.29-7.61 (m, 32H); ESI-MS: m/z calcd. for $C_{50}H_{37}O_2P_2$, $[M+H]^+$ = 731.22; found: 731.23; elemental analysis calcd. (%) for $C_{50}H_{36}O_2P_2$, C 82.18, H 4.97; found: C 82.40, H 4.76.

FT-IR(ATR) = 1188 (st, P=O) cm^{-1}.

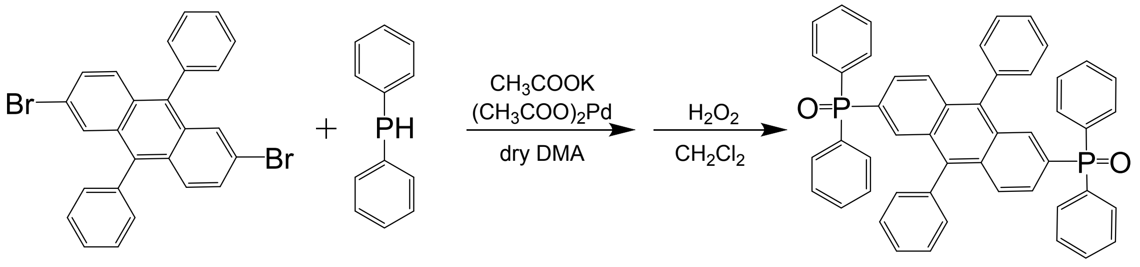

Scheme 1. Synthesis of **DPA-P** ligand.

Preparation of $[Lu(hfa)_3DPA\text{-}P]_n$ (Fig. S1): Methanol solution (0.5 mL) containing $Lu(hfa)_3(H_2O)_2$ (7 mg, 0.01 mmol) and CH_2Cl_2 solution (0.5 mL) containing DPA-P (200 mg, 0.35 mmol) were prepared, respectively. The methanol solution is added to microtube, and the CH_2Cl_2 solution is added to microtube slowly. Liquid-liquid diffusion-assisted crystallization from CH_3OH ($Lu(hfa)_3(H_2O)_2$)/ CH_2Cl_2 (DPA-P ligand) solution gave crystals.

Elemental analysis calcd. (%) for $C_{65}H_{39}F_{18}O_8P_2Lu$, C 51.13, H 2.57; found: C 50.82, H 2.44. FT-IR(ATR) = 1655 (st, C=O), 1249 (st, C-F), 1169 (st, P=O) cm^{-1}.

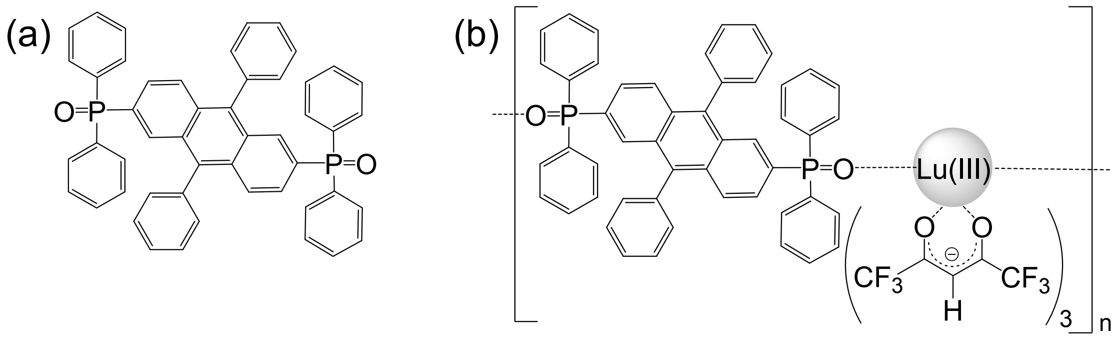

Fig. S1. Chemical structures of DPA-P (a) and $[Lu(hfa)_3DPA\text{-}P]_n$ (b).

Single-Crystal X-ray Structure Determination: X-ray crystal structures and crystallographic data for DPA-P and $[Lu(hfa)_3DPA\text{-}P]$ obtained by the recrystallization process using the CH_3OH ($Lu(hfa)_3(H_2O)_2$) / CH_2Cl_2 (DPA-P ligand) solvent system are shown in Fig. 2 and Table S1. X-ray crystal structures and crystallographic data for $[Lu(hfa)_3DPA\text{-}P]$ obtained by the recrystallization process using the $CHCl_3$ ($Lu(hfa)_3(H_2O)_2$) / CH_2Cl_2 (DPA-P ligand) solvent system is shown in Fig. S5 and Table S3. Single crystals of the compounds were mounted on micromesh (MiTeGen M3-L19–25L) using paraffin oil. Measurements were made by using a Rigaku RAXIS RAPID imaging-plate area detector or XtaLAB AFC11 (RCD3) with graphite-monochromated Mo-Kα radiation. Non-hydrogen atoms were anisotropically refined. All calculations were performed using a crystal-structure crystallographic software package. The CIF data were confirmed by the check CIF/PLATON service. CCDC-2046734 (for DPA-P), CCDC-2046736 (for $[Lu(hfa)_3DPA\text{-}P]_n$ obtained by the recrystallization process using the CH_3OH/CH_2Cl_2 solvent system), and CCDC-2058490 (for $[Lu(hfa)_3DPA\text{-}P]_n$ obtained by the recrystallization process using the $CHCl_3/CH_2Cl_2$ solvent system) contain the supplementary crystallographic data for this paper. These data can be obtained free of charge from The Cambridge Crystallographic Data Centre via www.ccdc.cam.ac.uk/data_request/cif.

S2. X-ray crystal data

Table. S1 Crystallographic data for DPA-P and $[Lu(hfa)_3DPA\text{-}P]_n$ obtained by the recrystallization process using the CH_3OH/CH_2Cl_2 solvent system.

	DPA-P	$[Lu(hfa)_3DPA\text{-}P]_n$
chemical	$C_{50}H_{36}O_2P_2$	$C_{65}H_{39}F_{18}LuO_8P_2$ [+CH_3OH]
formula weight	730.73	1526.87
crystal system	monoclinic	monoclinic
space group	$P\,2_1/n$	$P\,2_1/c$
a / Å	10.7593(3)	18.1201(3)
b / Å	10.8776(3)	13.7081(2)
c / Å	16.3514(4)	25.8676(4)
volume / Å^3	1851.62(9)	6415.04(17)
Z	2	4
density / g cm^{-3}	1.311	1.581
Temperature / °C	−150	−150
R	0.0407	0.0351
wR_2	0.1112	0.1282

S3. Shape measure calculation

To clarify the polyhedral structure, we calculated continuous shape measures. The continuous shape measure factor, S, was calculated to estimate the degree of distortion in the first coordination sphere of the coordination structure based on the crystal structure data. The S value is given by the following equation,

$$S = \min \frac{\sum_k^N |Q_k - P_k|^2}{\sum_k^N |Q_k - Q_o|^2} \times 100,$$

where Q_k represents the vertices of the actual structure, Q_0 is the center of mass of the actual structure, N is the number of vertices, and P_k represents the vertices of the ideal structure. Based on the shape measure calculation, $[Lu(hfa)_3DPA\text{-}P]_n$ was categorized as having SAP coordination geometry (S = 0.844).

Table S2. Shape measure calculation results

	SAP(D_{4d})	TDH(D_{2d})	BTP(C_{2v})
$[Lu(hfa)_3DPA\text{-}P]_n$	0.844	1.529	1.588

S4. The X-ray crystal structure views of the $[Lu(hfa)_3DPA\text{-}P]_n$ obtained by the recrystallization process using the CH_3OH/CH_2Cl_2 solvent system

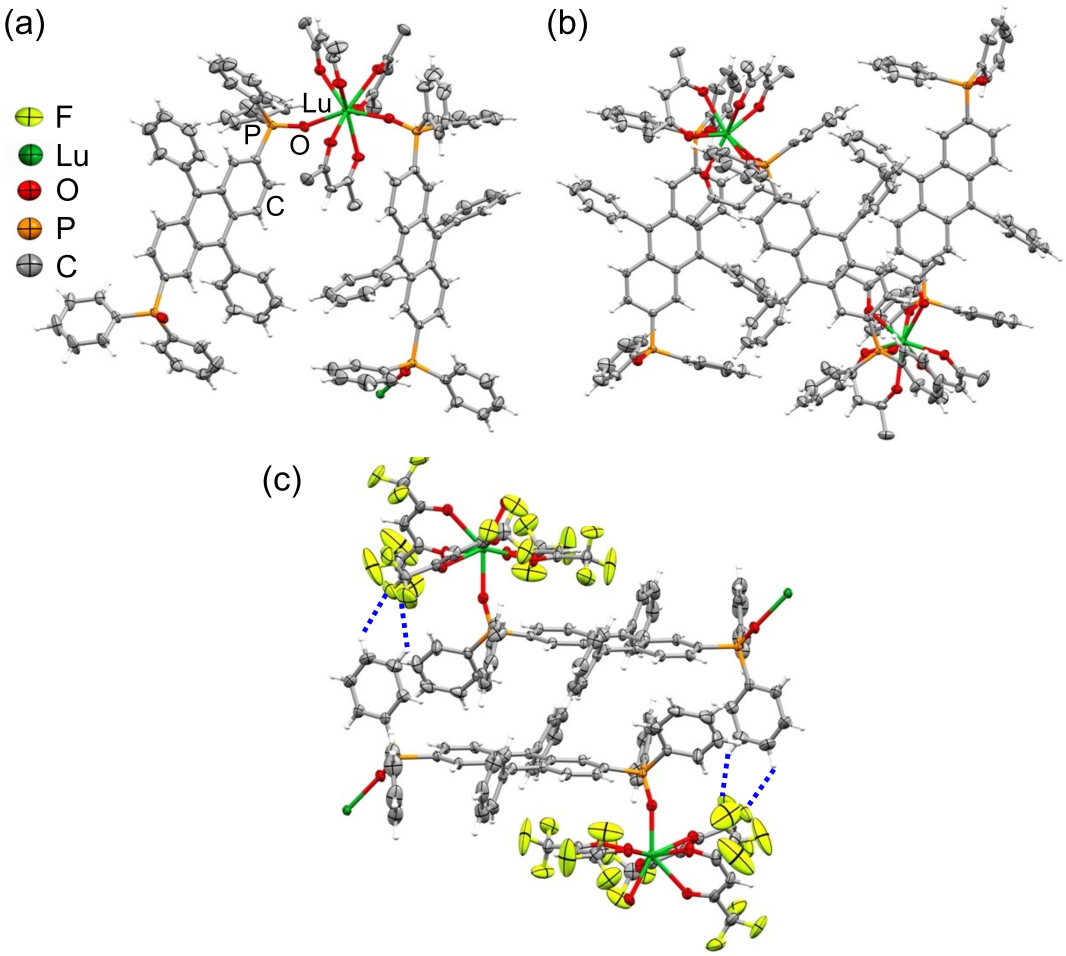

Fig. S2. The X-ray crystal structures of the $[Lu(hfa)_3DPA\text{-}P]_n$ obtained by the recrystallization process using the CH_3OH/CH_2Cl_2 solvent system (a: $Lu(hfa)_3(DPA\text{-}P)_2$ without F atom, b: $Lu_2(hfa)_6(DPA\text{-}P)_3$ without F atom; c: Packing structure in $[Lu(hfa)_3DPA\text{-}P]_n$ (Blue broken line: CH-F interaction).

S5. Emission lifetimes

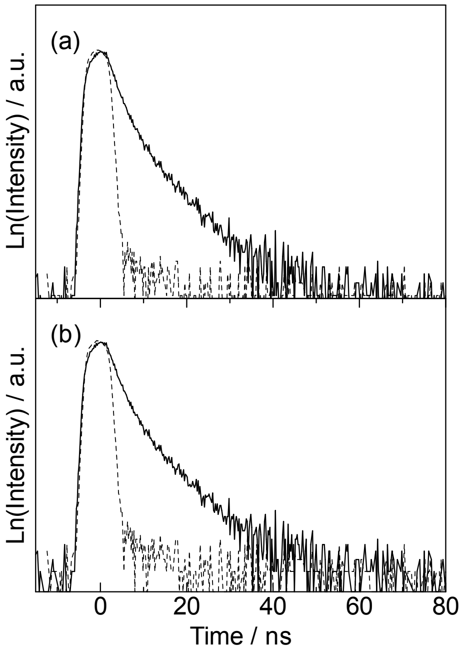

Fig. S3. Emission decay curves of DPA-P (a, solid line) and [Lu(hfa)$_3$DPA-P]$_n$ (b, solid line) obtained by the recrystallization process using the CH_3OH/CH_2Cl_2 solvent system, and prompt curves (a, b, broken line).

S6. CIE (x, y) chromaticity coordinate

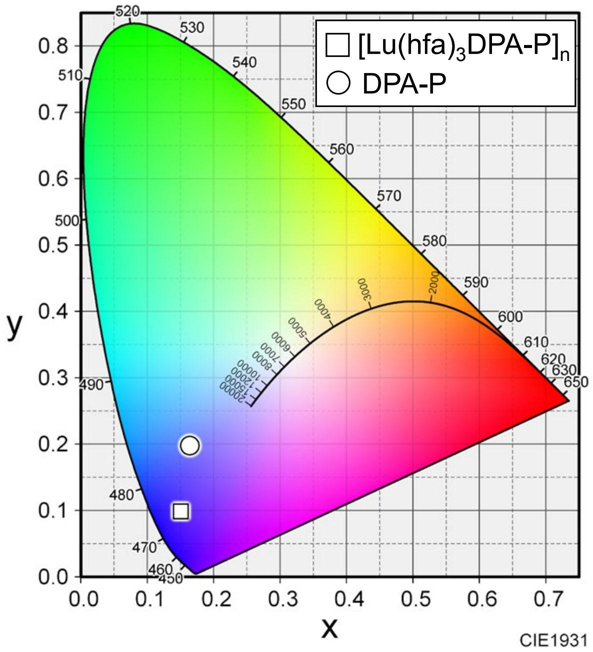

Fig. S4. CIE (x, y) chromaticity coordinates for DPA-P and [Lu(hfa)$_3$DPA-P]$_n$ obtained by the recrystallization process using the CH_3OH/CH_2Cl_2 solvent system

S7. Crystal structure of $[Lu(hfa)_3DPA\text{-}P]_n$ obtained by the recrystallization process using the $CHCl_3/CH_2Cl_2$ solvent system

We also prepared single crystals of $[Lu(hfa)_3DPA\text{-}P]_n$ by recrystallization from the $CHCl_3/CH_2Cl_2$ solvent system (Fig S5). The crystal was identified by elemental analysis ($C_{65}H_{39}F_{18}O_8P_2Lu$ calcd for C 51.13, H 2.57, found: C 50.87, H 2.47). The structure of the obtained crystal (Fig. S5, Table S3) is different from that of the single crystals obtained by recrystallization from the CH3OH/CH2Cl2 solvent system (Fig. 2b, Table S1). The structure of the $[Lu(hfa)_3DPA\text{-}P]_n$ crystal obtained from the $CHCl_3/CH_2Cl_2$ solvent system belongs to the $P2_1$ space group (Table S3). The CH–F interactions (< 3.0 Å) between the hfa unit and the aromatic DPA-P ligands were also observed in the $[Lu(hfa)_3DPA\text{-}P]_n$ (Fig. S5).

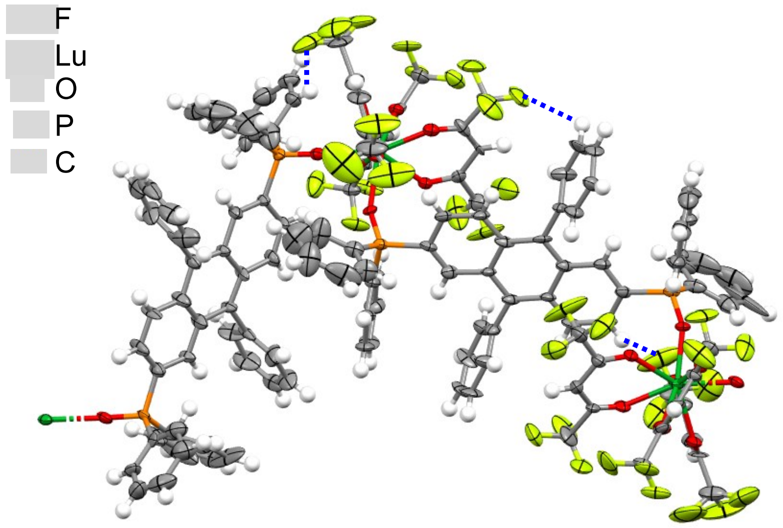

Fig. S5. X-ray crystal structure of $[Lu(hfa)_3DPA\text{-}P]_n$ obtained by recrystallization from the $CHCl_3/CH_2Cl_2$ solvent system.

Table. S3 Crystallographic data for $[Lu(hfa)_3DPA\text{-}P]_n$ obtained by the recrystallization process using the $CHCl_3/CH_2Cl_2$ solvent system.

	$[Lu(hfa)_3DPA\text{-}P]_n$
chemical	$C_{130}H_{78}F_{36}Lu_2O_{16}P_4$ [+$CHCl_3$]
formula weight	3053.75
crystal system	monoclinic
space group	$P\,2_1$
a / Å	21.8457(5)
b / Å	15.3407(2)
c / Å	23.4512(6)
volume / Å^3	7800.4(3)
Z	2
density / g cm^{-3}	1.394
Temperature / °C	−150
R	0.0530
wR_2	0.1417

The emission and excitation spectra are shown in Fig. S6. The FWHM is estimated to be 63 nm and is narrower than that of the DPA-P ligand (77 nm). The emission quantum yield upon excitation at 380 nm is estimated to be 30% and is higher than that of the DPA-P ligand (18%). The results also support our concept; the characteristic lanthanide fixation system improved the emission color purity and emission quantum yield.

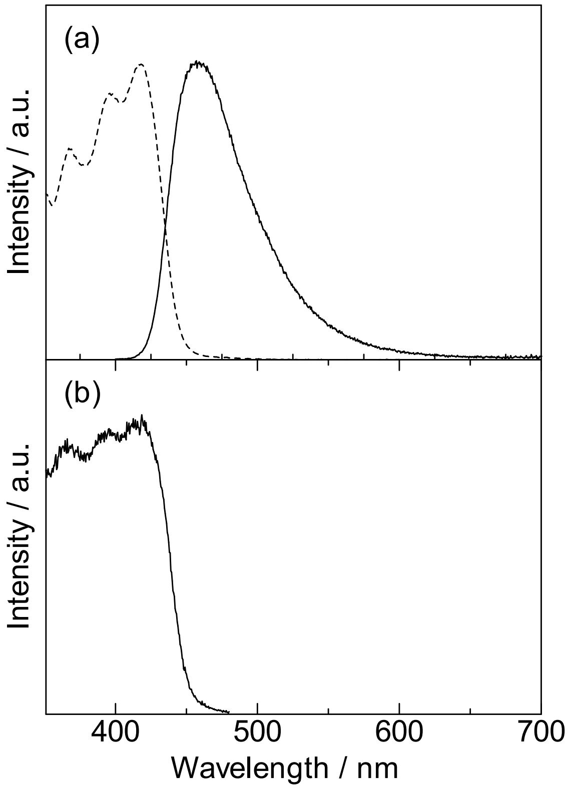

Fig. S6. (a) Absorption (broken line), emission (solid line), and (b) excitation (solid line) spectra of $[Lu(hfa)_3DPA\text{-}P]_n$ in their solid states, which is obtained by the recrystallization process using the $CHCl_3/CH_2Cl_2$ solvent system. The samples were 3000-fold dilution using KBr.
